# Making waves: how ultrasound-targeted drug delivery is changing pharmaceutical approaches

**DOI:** 10.1039/d1ma01197a

**Published:** 2022-02-23

**Authors:** Lauren J. Delaney, Selin Isguven, John R. Eisenbrey, Noreen J. Hickok, Flemming Forsberg

**Affiliations:** Department of Radiology, Thomas Jefferson University 132 S. 10th Street, Main 763 Philadelphia PA 19107 USA flemming.forsberg@jefferson.edu +1 (215) 955-4870; Department of Orthopaedic Surgery, Thomas Jefferson University, 1015 Walnut Street Philadelphia PA 19107 USA

## Abstract

Administration of drugs through oral and intravenous routes is a mainstay of modern medicine, but this approach suffers from limitations associated with off-target side effects and narrow therapeutic windows. It is often apparent that a controlled delivery of drugs, either localized to a specific site or during a specific time, can increase efficacy and bypass problems with systemic toxicity and insufficient local availability. To overcome some of these issues, local delivery systems have been devised, but most are still restricted in terms of elution kinetics, duration, and temporal control. Ultrasound-targeted drug delivery offers a powerful approach to increase delivery, therapeutic efficacy, and temporal release of drugs ranging from chemotherapeutics to antibiotics. The use of ultrasound can focus on increasing tissue sensitivity to the drug or actually be a critical component of the drug delivery. The high spatial and temporal resolution of ultrasound enables precise location, targeting, and timing of drug delivery and tissue sensitization. Thus, this noninvasive, non-ionizing, and relatively inexpensive modality makes the implementation of ultrasound-mediated drug delivery a powerful method that can be readily translated into the clinical arena. This review covers key concepts and areas applied in the design of different ultrasound-mediated drug delivery systems across a variety of clinical applications.

## Abstract 2

A review article regarding ultrasound-triggered drug delivery, highlighting exciting clinical trials and new applications for this modality.

## Graphical abstract

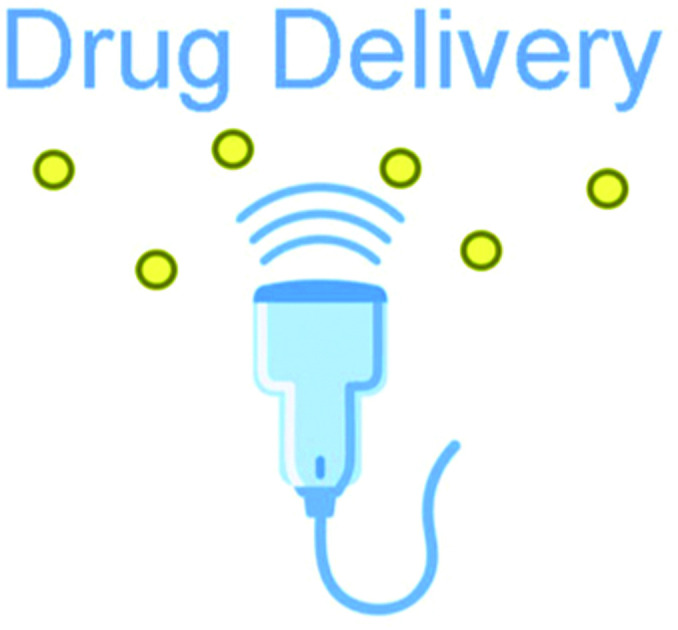

## Introduction

The delivery of drugs through oral and parenteral routes has enjoyed long success, but as disease treatments are refined, it is often apparent that a controlled delivery of drugs, either localized to a specific site or during a specific time, can increase efficacy and bypass problems with systemic toxicity. One approach that has increased delivery efficiency and therapeutic efficacy of drugs ranging from chemotherapeutics to antibiotics involves ultrasound technology improvements. These improvements often focus on increasing tissue sensitivity to the drug or can act as critical components of the drug delivery. In this review, we will focus on methods that allow non-invasive, spatiotemporal-specific drug delivery.

Ultrasound-targeted drug delivery (UTDD) builds upon the vast literature exploiting drug delivery systems. Specifically, local placement of drug elution systems, radioactive beads, *etc.*, has long been a mainstay in drug delivery systems.^1–3^ These first generation devices begin elution/decay from the moment of placement. However, drug concentrations are rapidly depleted, usually undergoing an exponential decrease in the concentration of the eluting drug. Such systems are exemplified by antibiotic-laden bone cement that is used to treat peri-prosthetic joint infections,^4^ antibiotic-impregnated coatings on penile implants,^5^ and transarterial chemoembolization,^6^ among others. Importantly, these drug delivery systems only achieve concentrations in the high therapeutic range for short durations—on the orders of hours and perhaps out to several weeks.^7^ Drug delivery devices of increasing sophistication aim for temporally- and spatially-controlled delivery of drugs, often with triggers that rely on a biological input.

To respond to this need for spatiotemporally controlled drug delivery, materials that release drug based on a response to a biological signal have been devised,^8,9^ and include pathway-sensitive,^10–12^ pH-sensitive,^13^ and even electrical^14^ triggering. However, all of these techniques are limited in clinical situations as they suffer from significant shortcomings associated with the transition from the well-defined *in vitro* to the complex *in vivo* environments. Prolonged drug release has been achieved through use of layer-by-layer coatings,^15^ where the duration of release can be tailored depending on the numbers of layers and their dissolution characteristics. Nonetheless, in all of these examples, the complete system is always implanted with limited or no external control.

To generate an external trigger, our group and others have turned to ultrasound waves. Ultrasound is a widely used modality for imaging and is selectively used for therapeutic applications such as extracorporeal shockwave lithotripsy, demonstrating safety and utility in the clinical arena.^16^ In terms of drug delivery, ultrasound allows precise temporal control of release, which is usually in real-time during the insonation period.^17^ In addition to temporal control, the high spatial resolution of ultrasound (on the order of mm) enables precise targeting, and the noninvasive, non-ionizing, and relatively inexpensive nature of ultrasound makes the implementation of UTDD more likely to be translated to clinical use.^18–20^

## Basics of ultrasound physics

Ultrasound imaging is characterized by the transmission of a short cyclic pressure wave (sound) (>20 kHz) from a transducer through the body. When transmitted waves passing through the tissues reach a boundary with an impedance mismatch, some of the energy (typically <1%) is reflected back to the transducer, while the remaining energy continues to pass through the tissue until it encounters another boundary or is absorbed by the body. Reflected waves are used to generate images. Some characteristics used to describe an acoustic signal/sound wave include its center frequency in MHz, acoustic pressure amplitude or peak negative (rarefactional) pressure in Pascals (Pa), pulse length in seconds, pulse repetition frequency in Hz, duty cycle, (percentage of time acoustic energy is actually being transmitted), and intensity in W cm^−2^.^21^ Clinical ultrasound, administered to patients, is also described by the mechanical index (MI), a safety index which relates to the risk of non-thermal damage to the tissue.^22^

Acoustic parameters can be manipulated to produce conditions that are conducive to UTDD by controlling frequency, acoustic pressure, intensity, radiation forces, and focal zone. The frequency of the sound wave determines (1) the depth of penetration, where lower frequencies allow imaging of deeper tissues; (2) imaging resolution, since higher frequencies provide higher resolution; and (3) degree of cavitation.^23,24^ Adjustments to the transducer will modulate the acoustic pressure and impact the amount of energy that is received by the imaging target. The sound wave is characterized by the peak positive pressure (*i.e.*, maximum compressional pressure) and the peak negative pressure, which is the minimum rarefactional pressure.^23,25^ Similar to acoustic pressure, the intensity of the ultrasound wave describes the amount of power carried by the wave through the medium to which it is applied, and is sensitive to the density of the target as well as the properties of the propagation medium.^23,26^ Intensity can be modulated through the characteristics of the ultrasound wave pulse (pulse length, pulse repetition frequency, and duty cycle).^27^ Radiation forces are those that occur between the ultrasound wave, target tissues, and/or target particles, and are neither thermal nor cavitational. When used with particles, the ultrasound wave produces a unidirectional force along the path of the beam that pushes the particles against the blood vessel walls, and the magnitude of that force is proportional to the acoustic frequency and intensity.^23,28^ Finally, ultrasound waves can also be administered in either a focused or a non-focused fashion. Historically, it was believed that it was best to use focused ultrasound as the highest intensity is found at the focal zone.^29^ Certain therapeutic techniques, such as lithotripsy and liver tumor and uterine fibroid ablation, now utilize high-intensity focused ultrasound (HIFU) in order to cause desired physiological changes in the target tissue.^30^ However, clinical practice generally uses diagnostic ultrasound with broader unfocused signals within the FDA limits that produce multiple reflections across a larger area of interest, avoiding unintended damaging bioeffects (*i.e.*, tissue damage) of a more focused, therapeutic, high intensity beam.^23,31^

**Fig. 1:**
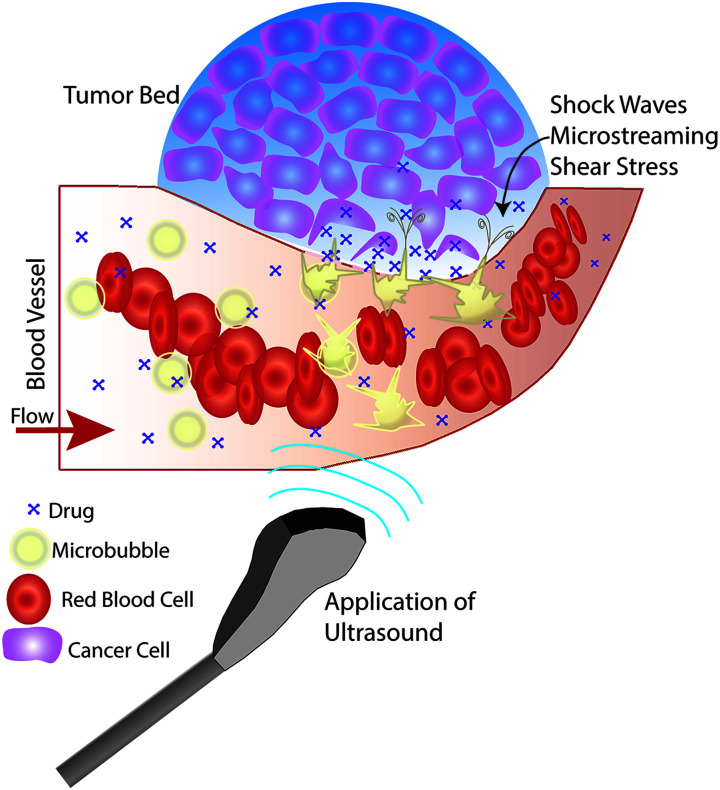
Effects of microbubble enhanced ultrasound on the local environment. The depicted scenario includes a tumor bed and an intravenous co-administration of microbubbles and drug, where ultrasound is applied to the local site of interest. Ultrasound induces cavitation of the microbubbles, resulting in physical effects such as shock waves, microstreaming, and shear stress. These effects disturb and eventually cause pore formation in the vessel wall and cell membranes as well as greater tumor cell separation, which allow drugs to penetrate deeper into the tumor bed.

The physiological effects induced by ultrasound insonation have been used in a variety of ways for targeted drug delivery. Studies of mechanical means for improving drug delivery (*i.e.*, ultrasound pressure waves) have demonstrated increased cytotoxicity and drug retention at the tumor site when exposed to therapeutic ultrasound *in vitro* (up to a 3-fold increase for doxorubicin (DOX)) compared to cells that were not insonated.^32,33^ Similarly, a limited number of studies have demonstrated increased antibiotic efficacy against infections using therapeutic ultrasound.^34–37^ Increased cytotoxicity is likely due to increased cell uptake of drug in response to ultrasound-induced increases in cell membrane permeability.^33,34,38^ However, augmenting drug delivery *via* ultrasound insonation is typically more efficacious when combined with microbubble-based ultrasound contrast agents (UCA) (Fig. 1); specifically, in the form of ultrasound-triggered microbubble destruction (UTMD) and sonoporation.^34,39,40^

## Microbubble cavitation-based drug delivery

One of the most well-known UTDD methods involves the use of microbubbles, which generally consist of a gas core encapsulated by a stabilizing shell usually made of lipid, protein, or polymer. Microbubbles, including micelles and liposomes, often are used as UCA to enhance image quality, but can also be used to augment or facilitate drug delivery within the body as a result of their unique interaction with and response to acoustic waves. Ideally, microbubbles used for UTDD would be non-toxic, be injected intravenously, have a diameter of 8 μm or less to pass freely through the capillary bed, provide acoustic enhancement, and have appropriate stability to withstand the duration of administration.^41^

**Fig. 2:**
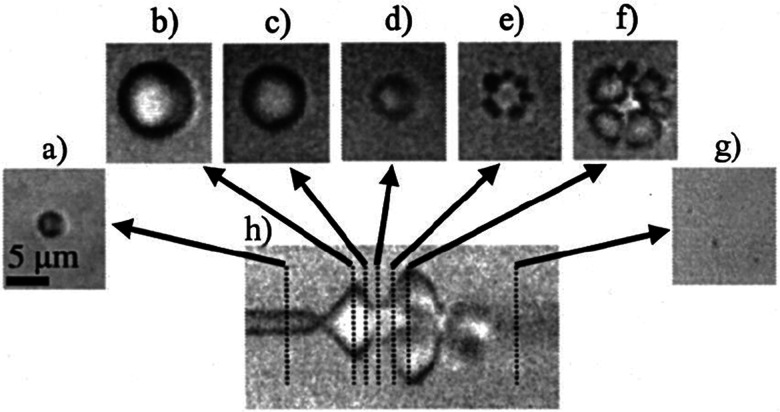
Optical frame (a–g) and streak (h) images showing the oscillation and fragmentation of an ultrasound contrast agent microbubble. The microbubble has an initial diameter of 3 μm (a), and the streak image (h) shows the changes in the bubble diameter as a function of time and the ultrasound pressure wave, with the fragmentation occurring during compression (e and f). Reprinted with permission from ref. 53.

Broadly, intravenously-injected UCA will reflect ultrasound, due to the acoustic impedance mismatch between blood and the gas encapsulated within the UCA (increasing contrast by more than 20 dB).^42,43^ The bubbles expand and contract as the acoustic wave passes, in a phenomenon known as cavitation.^44^ The degree to which the agent expands and contracts, or cavitates, is proportional to the pressure rarefaction and compression of the sound wave, respectively.^27,44^ Microbubbles generate three distinct modes of behavior in response to the ultrasound wave that can be utilized for therapeutic applications.^34,39,40,45^ At low acoustic powers (typically MI < 0.2), the ultrasound pulses used for imaging cause the bubbles to vibrate and this can cause small ruptures in cell membranes locally increasing the delivery of drugs, although not necessarily causing a regional increase in drug delivery.^39^ As the acoustic power increases, changes in vasculature permeability, caused by larger ruptures in capillaries leading to the escape of blood, pooling, and an increase in local drug uptake (Fig. 1), occur at MI values between 0.2 and 0.8.^45–49^ This phenomenon is known as sonoporation and is due to the UCA oscillations becoming nonlinear (so called stable cavitation) with limited microbubble disruption beginning to emit low-energy shock waves. This disruption increases markedly as the acoustic intensity is increased further (generally MI > 0.8) when the UCA readily burst producing copious high-energy shock waves emanating from the microbubbles (so called inertial cavitation).^39,45,46^ Cavitation and sonoporation are improved at lower ultrasound frequencies when the peak negative pressure is maintained, suggesting the importance of wave amplitude.^50–52^ Representative images of a cavitating and fragmenting microbubble are shown in Fig. 2.^53^

Disrupting and destroying microbubbles (*i.e.*, UTMD) have the potential to alter vascular structures such as cell junctions as well as cell membranes *via* mechanisms that include radiation force, shock waves, sonoporation, and microstreaming (*cf.*, Fig. 1).^34,39,45,54–56^ As the bubble collapses against a boundary, fluid will be focused and accelerated through the bubble, forming a liquid jet in the direction of the boundary that can travel over 20 μm with an average velocity of roughly 80 m s^−1^ and pressures up to 60 MPa.^44,57^ Thus, application of ultrasound will oscillate microbubbles present in the microcirculation and induce mechanisms that increase the local permeability of the vasculature, allowing the released or co-administered drugs to extravasate into the targeted tissue. Several mechanisms are known to induce such bioeffects, including sonoporation and endocytosis for intracellular delivery, disruption or reversible opening of the endothelial cell junctions, as well as modification of the fenestration pores or other alterations of the vascular endothelium.^45^ It is important to keep in mind that while UCA are typically delivered systemically, the ultrasound pulse can be targeted so that effects can be localized to the targeted tissue for a desired clinical application (*e.g.*, cancer treatments, blood brain barrier (BBB) opening, biofilm disruption, *etc.*).^34,45^

Over the last few decades, many iterations of microbubbles and UCA have been developed with various combinations of gases (*e.g.*, air, sulfur hexafluoride, and oxygen) and shell materials (*e.g.*, phospholipids, synthetic polymers, proteins, and surfactants) with mixed success.^43,58,59^ Currently, only three UCA (Optison (GE Healthcare), Definity (Lantheus Medical Imaging), and Lumason/SonoVue (Bracco)) are approved by the FDA for clinical use in the United States. However, there are numerous micro- and nano-scale agents at various stages along the research pipeline that can be used for UTDD. In addition to co-administration of microbubbles and drug, three general classes of microbubble technologies have been explored for drug delivery:^60^ (1) drug loaded microbubbles; (2) *in situ* formed microbubbles from nanodroplets; and (3) targeted microbubbles (*e.g.*, microbubbles with ligands attached for targeting to cell surface receptors). Therapeutic agents have also been loaded into microbubbles and UCA for drug delivery without systemic administration of free drug, aiming to increase bioavailability at the target site, while sparing healthy tissues from collateral damage from exposure to the drug.^27^ This concept relies on ultrasound pressures to induce inertial cavitation and UCA destruction at the target site, as discussed previously, resulting in drug release localized to the region of interest. However, due to the confined volume available for drug loading, the amount of drug that can be incorporated into a microbubble shell or membrane is limited.^60,61^ In addition, attaching or incorporating a drug into the UCA microbubble shell may alter the biological activity of the drug or the acoustic capabilities of the UCA.^61–63^

Lipid-based microbubbles and UCA currently dominate the UTDD literature, due to their ease of fabrication, flexibility and versatility, immediate release profile, and commercial availability.^64–66^ Briefly, these agents are comprised of a monolayer of lipids surrounding a gas core, where both the lipid and the gas can be selected to influence the behavior and loading of the agent.^64,67^ Liposomes are sphere-shaped vesicles that consist of one or more phospholipid bilayers surrounding an aqueous liquid core, while micelles are also sphere-shaped but comprised of a lipid monolayer that assembles itself when exposed to an aqueous solution. As an alternative to thin-walled lipid-based agents, a good deal of research is being directed toward the development of polymer-shelled microbubbles, since their thicker shells (100–400 nm) can increase drug loading, which may ultimately improve UTDD. These polymeric microbubbles can withstand higher ultrasound pressures than lipid-based microbubbles, with studies showing tolerance of over 0.54 MPa higher rarefactional pressure levels than phospholipid agents.^68^ Initial efforts involved the use of natural polymers, such as alginate and collagen, but have since largely transitioned to synthetic polymers such as poly(lactic-*co*-glycolic) acid (PLGA) and poly(lactic acid) (PLA).^69^ Briefly, fabrication of microbubbles with synthetic polymers generally involves emulsification of an aqueous phase with a polymeric solution (in an organic solvent), followed by spray drying or lyophilization,^70–72^ or involves a microfluidics approach to producing monodisperse microbubble populations.^73^ Aside from lipid- and polymer-based microbubbles, groups are also investigating other types of microbubbles and agents, such as protein- and bioprotein-shelled microbubbles, liposomes, and micelles, for UTDD applications.^74–76^

### Cancer-related delivery

**Fig. 3:**
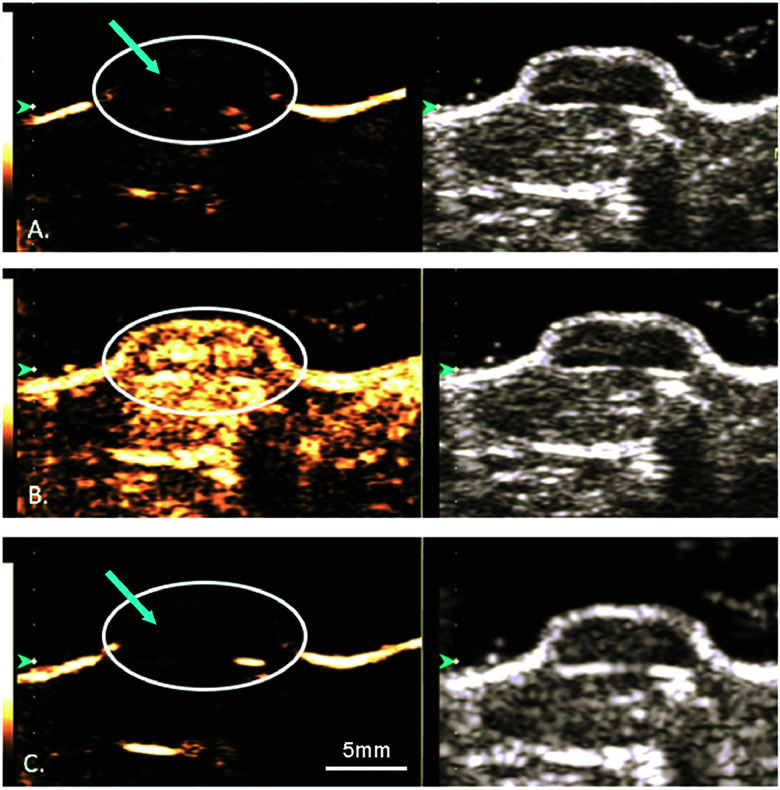
Ultrasound imaging in contrast enhanced (left) and B-mode (right) of a human pancreatic cancer xenograft mouse model. (A) Microbubbles loaded with GEM are visualized (blue arrow) within the tumor (white circle). (B) The microbubbles and tumor are subjected to a 4 second, 1.35 MI destructive pulse to induce inertial cavitation and UTDD. (C) An absence of microbubbles is seen within the tumor, suggesting the destruction of the GEM-loaded microbubbles following the high-MI ultrasound pulse. Reprinted with permission from ref. 61.

Microbubbles have been studied as a targeted delivery vehicle for several types of cancers, with many recent review articles eloquently displaying the breadth of research in this area.^34,77–80^ This review will touch briefly on several recent efforts, including UTDD and sonoporation techniques. Wu *et al.* investigated the efficacy of pluronic polymer micelles, specifically P123/F127, to generate curcumin-carrying polymeric micelles for UTDD.^81^ Application and insonation (1.9 MHz, 0.4 W cm^−2^, 1 min) of these micelles resulted in significant inhibition of 4T1 breast cancer tumor growth with a 6.5-fold reduction in tumor weight compared to controls in a mouse model.^81^ Combining the concepts of immune shielding and multi-modal targeting, Jablonowski *et al.* decorated the surface of DOX-loaded PLA microbubbles with tumor necrosis factor-related apoptosis inducing ligand (TRAIL) and polyethylene glycol (PEG).^63,82^ These microbubbles demonstrated augmented tumoricidal activity after 20 minutes of insonation (5 MHz, 0.94 MPa) against MDA-MB-231 (up to 80%) and MCF7 (up to 60%) breast cancer cells in culture, while also achieving at least 30% decrease in immune activation.^63,82^ Recently, this group also showed some success in encapsulating gemcitabine within a PLA microbubble for treatment against MiaPaCa-2 pancreatic cancer cells.^61^ However, the *in vitro* success did not translate into tumor suppression in a mouse model (Fig. 3), highlighting the limitations of drug loading capacity within microbubbles.^61^ In an effort to improve drug loading and delivery, Teraphongphom *et al.* demonstrated the feasibility of loading quantum dots, magnetic iron oxide nanoparticles, and gold nanoparticles within the shell of PLA microbubbles, suggesting that these modalities could provide opportunities for multimodal imaging techniques or therapeutics.^83^

**Fig. 4:**
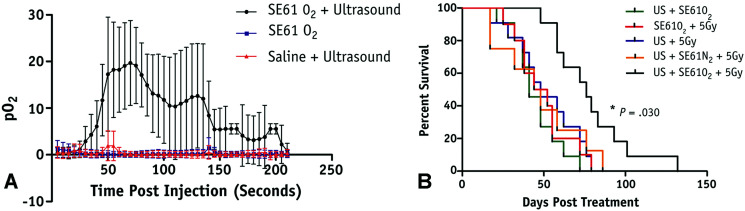
Results from oxygenation study using UTMD of oxygen loaded microbubbles. (A) Average intratumoral oxygenation profiles, demonstrating sustained *p*O_2_ elevation when SE61O_2_ microbubbles are insonation (black circles), but minimal changes in *p*_O2_ levels with insonation of saline injection (red triangles) or SE61O_2_ microbubbles without insonation (blue squares). Data points represent mean values, with error bars representing standard deviation. (B) Mouse survival in days following treatment, where mice treated with SE61O_2_ bubbles, ultrasound, and 5 Gy radiation (black line) had significantly increased radiation sensitivity and overall survival. Reprinted with permission from ref. 85.

Microbubbles can also be used to carry other factors, such as genes or gases, that can be used to improve cancer treatments. For example, Du *et al.* adopted an approach for gene delivery similar to those used for drug delivery by conjugating magnetic mesoporous silica nanoparticles carrying pGCMV/eGFP plasmid DNA to dipalmitoylphosphatidylcholine (DPPC)/1,2-distearoyl-*sn-glycero*-3-phosphoglycerol (DSPG)/*N*-(carbonyl-methoxypolyethyleneglycol 2000)-1,2-distearoyl-*sn-glycero*-3-phosphoethanolamine (DSPE-PEG2000) microbubbles.^84^ In a xenograft mouse model of ovarian cancer, this approach not only protected the vector from degradation but also protected surrounding cells from non-specific cytotoxicity from the vector. Additionally, they were able to maintain the cavitational properties of the microbubble for UTDD when insonated at 7 MHz with 0.6 MPa and 30% duty cycle for 1 minute.^84^ Eisenbrey *et al.* designed ultrasound-sensitive oxygen-loaded microbubbles stabilized by Span 60 and water-soluble vitamin E surfactant (SE61 O_2_) for targeted delivery of oxygen to hypoxic breast tumors *via* insonation (4.2 MHz, 2.5 MPa, 4 seconds) in a mouse model.^85,86^ By increasing the local tumor oxygenation by 20 mmHg immediately prior to radiation therapy, they achieved almost triple the amount of tumor radiosensitivity compared to the hypoxic controls (Fig. 4).^85,86^ This group also demonstrated that these SE61 O_2_ microbubbles were effective against brain metastases from these breast cancer models, suggesting a wide applicability of these agents.^87^ Building upon this work, this group has also investigated loading lonidamine within these oxygen-carrying microbubbles to achieve multi-modal UTDD against hypoxic tumors, including breast and head and neck cancers.^88,89^

Significant efforts have also been devoted to sonoporation of pancreatic ductal adenocarcinoma (PDAC), since this remains a lethal cancer with an overall five year survival rate of approximately 10% and a median survival for all patients of less than 6 months.^90,91^ Standard-of-care chemotherapy regimens have only marginally increased overall survival rates, and thus, are largely ineffective in preventing recurrence and eventual death.^92,93^ Recently, Logan *et al.* developed a novel approach to loading a combination of drugs within a lipid-based microbubble by creating a drug-laden phospholipid molecule for use in a one-pot synthesis of a dual-loaded UCA.^94^ They combined a solution of chloroform and 1,2-dibehenoyl-*sn-glycero*-3-phosphocholine (DBPC) with another solution of gemcitabine, phospholipase D, and calcium chloride to generate a gemcitabine-carrying phospholipid complex that can be used to form the shell of a lipid microbubble.^94^ The final microbubble consisted of the lipid-gemcitabine molecule, DSPE-PEG2000, and paclitaxel (5 : 1.43 : 2.5 weight ratio) surrounding a perfluorobutane core, and resulted in a 40% reduction in tumor growth in a BxPC-3 pancreatic cancer mouse model when administered in conjunction with ultrasound (1 MHz, 3.5 W cm^−2^, 0.48 MPa).^94^ In another approach, Dwivedi *et al.* designed a DOX-loaded magneto-liposome-ligated microbubble complex, where the iron oxide nanoparticles were loaded with DOX then encapsulated within oligolamellar vesicles, which were then covalently conjugated to lipid-based microbubbles (1,2-distearoyl-*sn-glycero*-3-phosphocholine (DSPC))/cholesterol/DSPE-PEG2000-maleimide (60 : 20 : 20 molar ratio) with a perfluorooctane gas core.^95^ The resulting magneto-liposome-ligated microbubble complex improved pancreatic tumor targeting and tumoricidal activity (approximately 80% reduction in tumor volume) in a pancreatic cancer xenograft mouse model.^95^ Additionally, groups are working to utilize ultrasound to enhance the permeability and drug uptake of PDAC tumors. Using xenograft mouse models of PDAC, Schultz *et al.* investigated PDAC sonoporation using all four worldwide clinically-approved UCA (Optison, Definity, Lumason/SonoVue, and Sonazoid (GE Healthcare)) and two ultrasound regimens (2.0 MHz 20 μs pulses at low and high acoustic powers for 10 minutes) to identify the ideal parameters to increase therapeutic efficacy.^96^ Treatment with high power ultrasound (*I*_SPTA_ = 200 mW cm^−2^) utilizing Sonazoid as the UCA most consistently caused an increase in permeabilization across different experiments and markers of permeability. The results from this and other pre-clinical studies indicate that UCA weaken endothelial cell junctions, increase fenestration sizes, and can generate minute resealing pores in cells, all of which can facilitate deeper penetration of chemotherapeutic agents into the PDAC tumor.^48,49,96^ A Phase I clinical trial aimed at augmenting standard-of-care chemotherapy efficacy led to significant improvement in 10 PDAC patients receiving sonoporation treatment compared to 63 historical controls.^97,98^ The results included tumor regression in 50% of the patients and a significant increase in the number of treatments patients were able to undergo (8.3 ± 6.0 cycles in controls, 13.8 ± 5.6 cycles after sonoporation treatment; *p* = 0.008). Most importantly, overall survival improved from 8.9 months in the controls to 17.6 months (*p* = 0.011).^97,98^ A larger, Phase II clinical trial is currently underway.^99^

### Infection-related delivery

Research on ultrasound-triggered enhancement of antimicrobials to treat infections is an emerging area for UTDD use.^34^ Recently, Horsley *et al.* demonstrated over 16 times greater intracellular delivery of gentamicin to an *in vitro* human bladder organoid model of urinary tract infection when their DSPC/DSPE-PEG/DSPE-PEG-biotin/1,2-dipalmitoyl-*sn-glycero*-3-phosphoethanolamine-*N*-(lissamine rhodamine B sulfonyl) (79.5 : 10 : 10 : 0.5 molar ratio) microbubbles decorated with gentamicin-loaded DSPC/DSPE-PEG liposomes when exposed to ultrasound (1.1 MHz, 2.5 MPa, 20 s) compared to uninsonated controls.^100^ Furthermore, twice the amount of delivery was achieved with liposomes that were insonated but not conjugated to microbubbles.^100^ A different application for UTDD that has recently been investigated is improving acute transplant rejection, such as the study of cardiac transplant rejection described by Liu *et al.*^101^ This group developed a DSPC/DSPE-PEG2000 (9 : 1 molar ratio) microbubble loaded with FK506 anti-rejection drug, and demonstrated both increased drug delivery (1.64-fold higher) and reduced cardiac graft rejection (average 4 day prolonged graft survival) in the group of rats that received both the loaded microbubbles and insonation with HIFU (1 MHz, 2 W cm^−2^).^101^

**Fig. 5:**
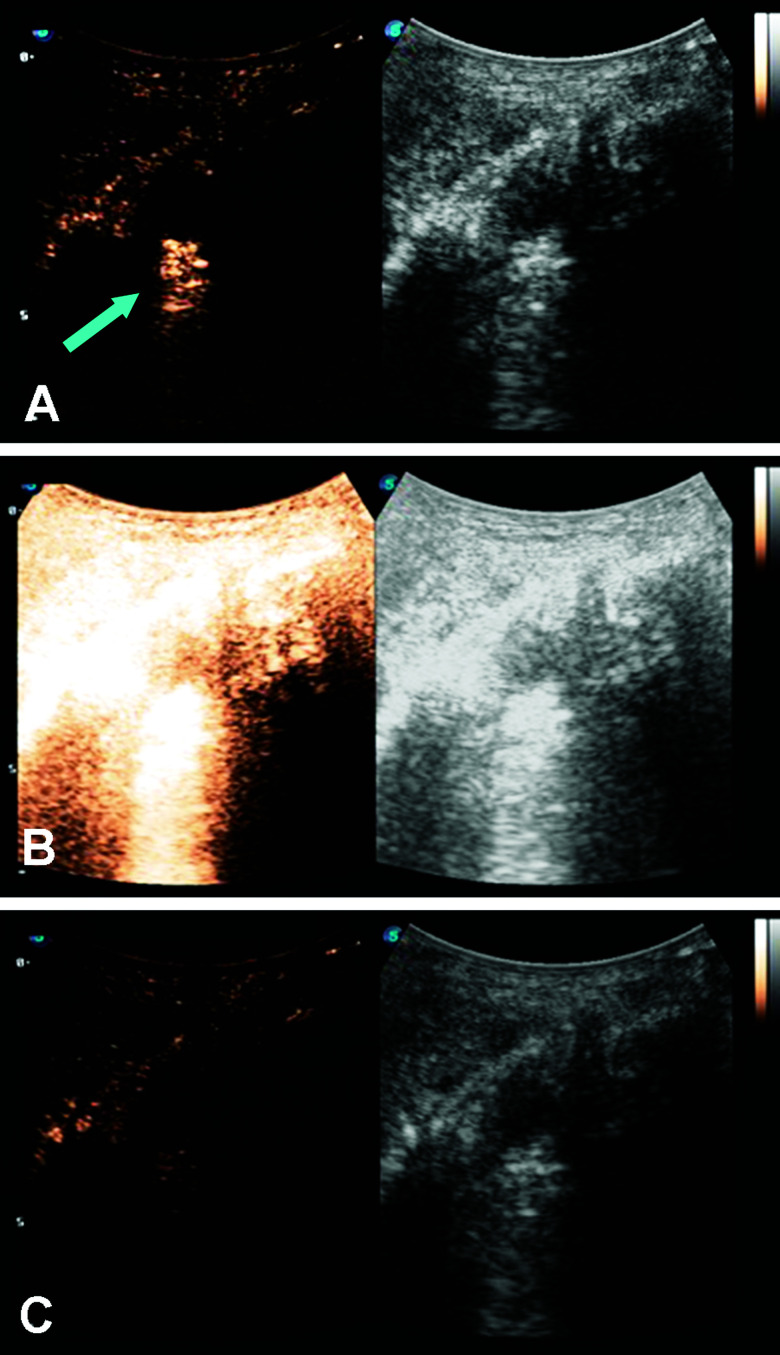
Ultrasound imaging in contrast enhanced (left) and B-mode (right) of UTMD in synovial fluid within a septic knee joint in a pig. (A) Microbubbles are visualized (blue arrow) within the septic joint. (B) The microbubbles and joint are subjected to a destructive pulse for UTMD. (C) An absence of microbubbles is seen within the septic joint, suggesting UTMD and UTDD following the destructive pulse.

Studies have shown *in vitro* and *in vivo* that 5–10 minutes of UTMD (80–300 kHz at 0.5–1.0 W cm^−2^ and a 50% duty cycle, which is well outside the limits of clinical scanners) *via* cavitation causes simple biofilms and catheter-associated biofilms to become more susceptible to vancomycin.^35,36,102^ The rupture of UCA microbubbles subjects biofilms to powerful shockwaves, which transiently permeabilize the cell membrane, increase antibiotic transport, increase biofilm extracellular matrix porosity, and decrease biofilm thickness.^102^ While joint infection rates have been improving, more than 20 000 patients per year will be diagnosed with bacterial infections of their synovial fluid.^103^ Such infections are a significant cause of morbidity and mortality, and the presence of large (often 2–5 mm in size), proteinaceous, bacterial biofilm aggregates may be a primary reason for joint infection antibiotic treatment failures.^103^ We tested the clinical applicability of injections of amikacin as well as Definity microbubbles together with application of UTMD in a pilot study of *Staphylococcus aureus* septic arthritis in 12 pig femorotibial joints (Fig. 5).^104,105^ Septic joints that received treatments of amikacin alone or of amikacin with ultrasound insonation (*i.e.*, without microbubbles) did not resolve the infection. When UTMD was applied concomitantly with injection of amikacin (4 s destructive pulses at an MI > 0.6 at 3.5–5.0 MHz), all five pigs with confirmed infection showed reduction of bacterial burden to below detectable levels (*p* = 0.008). This suggests that administration of antibiotics with microbubble cavitation could be an efficacious treatment approach to difficult cases of septic arthritis.^104,105^

### Other application areas

**Fig. 6:**
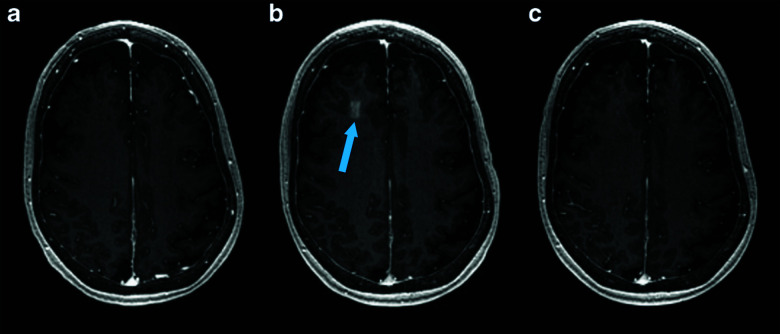
MRI images from baseline (a), immediately following HIFU (b), and 24 hours after HIFU (c), demonstrating opening and closure of BBB. Extravasation and pooling of contrast within the insonated portion of the frontal lobe is visible immediately following HIFU administration (b, blue arrow) demonstrating opening of the BBB, but is absent 24 hours later (c) suggesting BBB closure. Reprinted (modified) with permission from ref. 116.

While the BBB maintains the homeostasis of the brain, it also blocks over 98% of small drugs (<600 Da) and all larger therapeutic molecules from entering the brain, limiting the applicability of a large number of drugs.^106,107^ Focused ultrasound (with peak negative pressures >200 kPa and transmit frequencies <2 MHz) and UCA microbubbles (most frequently Definity) have been used to transiently increase the permeability of the BBB in various pre-clinical settings both paracellularly and transcellularly, including more recently in primates.^108–111^ Omata *et al.* investigated whether the encapsulated gas core had an effect on the *in vivo* contrast and cavitation behavior of drug-loaded lipid microbubbles composed of DSPC, DSPG, and DSPE-PEG2000 (30 : 60 : 10 molar ratio) during circulation in a mouse model.^112^ In their study, perfluoropropane and perfluorobutane were most effective at retaining contrast properties as well as delivering Evans blue dye as a model drug to the brain when the agents were insonated at 3 MHz (0.5 W cm^−2^ intensity) for 3 minutes.^112^ Interestingly, Shekhar *et al.* demonstrated effective encapsulation and ultrasound-triggered (6 MHz Doppler, MI 0.8 and 220 kHz pulsed, MI 0.47, 10 seconds) delivery of xenon to the brain in a mouse model using a DSPC/DSPE-PEG2000 (9 : 1 ratio) lipid-shelled microbubble loaded with xenon and octafluoropropane, to help reduce and stabilize neurologic injury in stroke.^113^ Most excitingly, since 2018, several small (4–5 patients per study) phase I safety and feasibility studies on permeabilizing the BBB in humans using focused ultrasound techniques have been reported with no serious clinical or radiological side effects.^114–116^ A representative image from one of these studies, demonstrating visible BBB opening, is shown in Fig. 6. In all three trials, an ultrasound device with 1024 individual transducers operating at a frequency of 220 kHz (ExAblate Neuro; InSightec) and power ranging from 5.2 to 19.4 W were used in conjunction with Definity microbubbles (4 μL kg^−1^). These studies pave the way for future large-scale human clinical trials to investigate efficacy of these methods for ultrasound-augmented drug delivery to the brain.

Wound healing applications are also gaining attention in the field of UTDD. For example, Zhu *et al.* designed a paclitaxel-loaded lecithin/cholesterol (3 : 1 weight ratio) microbubble *via* film hydration for targeted delivery to a rabbit iliac balloon injury model, demonstrating nearly doubled drug release (1 MHz, 1.5 MPa, 10 seconds) and a 30% reduction in vascular smooth muscle cell viability in the model.^117^ Similarly, Wang *et al.* showed a 10-fold improvement in drug penetration in stented coronary artery tissue in response to low-intensity pulsed ultrasound (10–900 kHz, with 0.1–10% acoustic power) of surfactant-stabilized microbubbles embedded with paclitaxel-loaded PLGA nanoparticles.^118^ They showed a 10-fold increase in drug retention in a porcine coronary artery model, providing a good basis for continued research into precision delivery of antiproliferative drugs to stented vascular tissue.^118^ In a theranostic approach, Zhang *et al.* designed a PLGA microbubble loaded with simvastatin, and further conjugated their surface with anti-ICAM-1 antibodies that selectively bind to the target site, in order to evaluate and treat atherosclerosis, showing success with UTDD (450 W positive pressure amplitude) in a rabbit model.^119^ UTDD has also been investigated for delivering nitric oxide to sites of vascular injury, where nitric oxide is encapsulated within a microbubble to protect the gas from endogenous scavengers until it is released at the target site.^120^ For example, Tong *et al.* investigated whether insonation (1 MHz, 1 W cm^−2^, 60 seconds) of co-injected mesenchymal stem cells and nitric oxide-carrying 1,2-dipalmitoyl-*sn-glycero*-2-phosphoethanolamine-*N*-[methoxy (polyethylene glycol)-2000] (DPPE-PEG2000) lipid-based microbubbles could help treat myocardial infarction in a rat model.^121^ They demonstrated both enhanced mesenchymal stem cell transplantation efficiency (up to 4-fold) as well as increased angiogenesis (up to 2.5-fold) in response to insonation.^121^ Additionally, Kim *et al.* showed successful recovery of luminal area in a rat model of vasospasm following subarachnoid hemorrhage by insonating (1 MHz, 0.3 MPa, 60 seconds) echogenic liposomes (DSPE, 1,2-dioleoyl-*sn-glycero*-3-phosphocholine, and cholesterol, 60 : 30 : 10 ratio, plus 6% DPPE-PEG2000) loaded with nitric oxide and argon in a 1 : 9 ratio.^122^

Studies have shown that small molecules, such as genes and peptides, can be effectively delivered to the intracellular space when administered with UCA and focused insonation causing cavitation-induced permeability.^27,123–125^ For example, Dewitte *et al.* describe the development of mRNA-loaded lipid microbubbles with a perfluorobutane core stabilized by a DPPC and DSPE-PEG3400-biotin shell, and demonstrated the ability to visualize these carriers using contrast ultrasound in a canine model, suggesting the potential for these agents to be used for UTDD applications in gene therapy.^126^ Also, a new emerging technology is the development of lipoplexes and polyplexes, where lipid-based microbubbles can be ligated with a complex of nucleic acid and liposome or polymer, respectively, in order to load and deliver nucleic acids and take advantage of ultrasound-induced transfection enhancement.^127,128^

In another application, lysozyme-shelled microbubbles carrying minoxidil combined with insonation (1 MHz, 3 W cm^−2^, 0.266 MPa) enhanced hair follicle growth in a mouse model.^129^ They demonstrated approximately 66% increased hair follicle growth rates using the minoxidil-loaded lysozyme microbubbles with ultrasound, which was significant compared to control groups.^129^ Similarly, Liao *et al.* used ultrasound-sensitive lysozyme-shelled microbubbles to deliver adapalene to a mouse model of photoaging skin, resulting in significant wrinkle reduction with in the mice treated with this UTDD agent and insonation (1 MHz, 3 W cm^−2^ for 1 minute).^130^

Microbubbles and ultrasound have also been investigated for applications in drug delivery to the eye, including temporary disruption of the blood–retina barrier, increased cellular uptake, and targeted delivery.^131^ For example, Thakur *et al.* have recently developed ultrasound-responsive rhodamine-tagged nanobubbles that, when exposed to ultrasound (1 MHz, 0–2.5 W cm^−2^, 60 seconds), significantly increased the penetration depth (up to 5-fold) into *ex vivo* bovine and porcine eyes.^132^ Also, Du *et al.* achieved 18% trans-retinal siRNA transfection efficiency (6-fold increase compared to control) using UTMD (1 MHz, 2 W cm^−2^, 5 minutes) in a rat model.^133^

Notably, for all studies discussed, cellular uptake and drug efficacy was significantly increased when ultrasound-induced microbubble cavitation was included in the delivery method, further supporting the importance of insonation for improving drug delivery.

## Phase-change agents for delivery

Phase-change agents are stabilized liquid emulsions that, when insonated with certain ultrasound parameters, undergo a phase-transition to a gaseous state accompanied by a volumetric expansion through a process known as acoustic droplet vaporization (ADV).^134^ ADV is similar to cavitation of gaseous microbubbles *via* ultrasound, but there are some key differences. ADV involves liquid bubbles with boiling points near or below body temperature, with perfluorocarbons (PFCs) being a popular choice.^135^ These bubbles transition from liquid to gas with insonation depending on the vapor pressure of the liquid and the temperature achieved by the insonation.^136^ As the ultrasound frequency and Laplace pressure decrease and the duty cycle and peak negative pressure amplitude increase, the ADV events will increase.^135^ Such acoustic droplets were initially engineered because of the limitations of microbubbles, which are too large to cross blood vessel walls, whereas acoustic droplets are submicron in scale and have more extensive access. These advantages of acoustic droplets are highly valuable for therapeutic avenues; for example, these particles may penetrate deep into a tumor mass for drug delivery.^136^ Nanoemulsion-based ADVs, which are kinetically stable dispersions of two immiscible oil and water phases together with surfactant molecules, are another emerging technology with applications in UTDD.^137^ These locally-created microbubbles are then subject to cavitation, promoting ultrasound-triggered release of any encapsulated drug.^137^

Another particular phase-change-based approach to UTDD aiming to overcome the limitations of conventional systems is Acoustic Cluster Therapy (ACT).^138^ In this technique, a dispersion of microbubble/microdroplet clusters is formed by reconstituting the UCA microbubble with liquid oil microdroplets and then injected intravenously.^138^ The microdroplet oil component is designed to have a relatively low boiling point (<50 °C), with an ensuing high vapor pressure at body temperature, and low solubility in blood (<1 × 10^−4^ M). In one example, ACT was achieved by reconstituting Sonazoid along with perfluoromethylcyclopentane microdroplets stabilized with a DSPC membrane.^138^ Upon activation by low power ultrasound (MI < 0.4), the microdroplets transition into their gas phase and transfer the gas to the microbubble, forming larger bubbles that transiently lodge themselves in the microvasculature. Further insonation of the large (>20 μm) activated bubbles induces biomechanical effects, due to their close contact with the endothelial wall, and improves drug delivery.^138^

Phase change agents can also be incorporated into a matrix, such as a hydrogel, for increased stability and spatiotemporal control of release. Hydrogels are three-dimensional, cross-linked networks of water-soluble polymers that offer tunable physical and chemical properties, allowing a wide range of options for drug loading and delivery.^139–141^ The drug contained within the system can be incorporated so that it is freely available throughout the matrix, contained within particles that are themselves uniformly dispersed through the matrix, or exist as a gradient (*e.g.*, the drug particles localized in the center of the depot). One particular area of interest using hydrogel-based UTDD is acoustically-responsive scaffolds (ARSs), which are hydrogels that house emulsions responsive to ADV.^142,143^

### Cancer-related and infection-related delivery

Phase-change agents have been explored for disrupting bacterial biofilms as well as cancerous tissues. Other phase-change contrast agents (typically liquid PFC droplet stabilized by a phospholipid shell) with 100–400 nm diameters have also been used to enhance antibiotic efficacy against biofilms by up to 94%.^37^ Rapoport *et al.* describe development of ultrasound-activated, paclitaxel-carrying, PEG-PLLA nanoemulsions that convert into microbubbles upon insonation (1 MHz, 3.4 W cm^−2^) for cancer treatment. This ultrasound-triggered nanotherapy causes tumor regression by an order of magnitude in ovarian, breast, and orthotopic pancreatic cancer mouse models.^144^ Additionally, de Matos *et al.* demonstrated effective UTDD (1.3 MHz, 2–24 MPa, 1 minute) of mistletoe lectin-1 against a mouse model of colon cancer using a PFC nanoemulsion encapsulated within DPPC/cholesterol/DSPE-PEG liposomes.^145^ The ACT concept has been used in combination with Abraxane, DOX, and irinotecan to successfully treat subcutaneous tumors of human prostate, breast, and colon cancer, respectively, in mice.^146–148^ Recently, it was announced that a Phase I trial of ACT (*i.e.*, Sonazoid conjugated with liquid oil microdroplets) in patients with liver metastases of gastrointestinal origin is underway in the United Kingdom.^149^

### Other applications

**Fig. 7:**
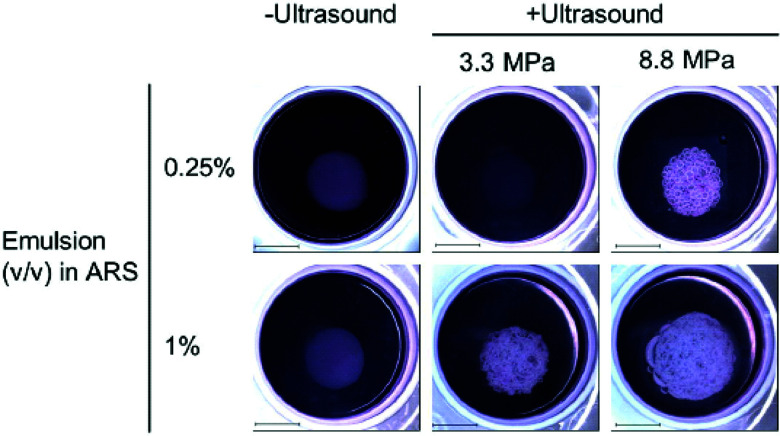
Results from insonation of the gel-in-gel ARS construct for UTDD of basic fibroblast growth factor at 7 days post-insonation. Macroscopic bubble formation was observed in all insonated samples except the 0.25% emulsion exposed to 3.3 MPa, and no bubbles were formed in the absence of ultrasound. Scale bar = 2 cm. Reprinted (modified) with permission from ref. 150.

Phase-change agents have also been used for angiogenesis and wound healing, as well as temporary disruption of the BBB for enhanced drug delivery. For example, promotion of angiogenesis was achieved with UTDD using HIFU (2.5 MHz) of a basic fibroblast growth factor payload housed within a water-in-PFC-in-water emulsion encased in a fibrin-based ARS that also contained endothelial cells and fibroblasts surrounding the payload core (0.25% and 1% v/v).^150^ For endothelial network formation, both a higher volume of growth factor-loaded emulsion and acoustic pressure improved results (Fig. 7).^150^ Total tubule length was statistically longer for the 1% growth factor-loaded emulsion *vs*. the 0.25% formulation at both 3.3 MPa and 8 MPa, and total tubule length was also longer for the 8.8 MPa samples for both the 0.25% and 1% emulsions compared to their 3.3 MPa counterparts.^150^

Another study by this group looked at single and dual drug release, using basic fibroblast growth factor and platelet-derived growth factor with dextran payloads, *via* ADV from fibrin-based ARSs using standing wave field ultrasound (2.5 MHz for single payload, 3.25 MHz and 8.6 MHz for dual payload).^142^ In this study, the possibility of staged drug delivery was important because of the temporal control of angiogenesis by different growth factors.^142^ The use of a standing wave field produced elevated amplitudes through constructive superposition without using high acoustic input energies. The authors found that higher excitation frequencies were better suited for spatial selectivity with the downside of prolonged insonation period. ACT has also been used to safely and temporarily open the BBB, using an acoustic power 5–10 times lower than those applied for conventional microbubbles (*i.e.*, within the diagnostic range).^151^ Using these conditions, small (Gadodiamide, 591.67 Da) and large (IRDye 800CW-PEG, ∼45 kDa) molecules have been successfully delivered into the brain.^151^ Interestingly, Wu *et al.* utilized lipid-based nanodroplets with octafluoropropane and decafluorobutane cores without an additional carrier to disrupt and cross the BBB to deliver protein-sized molecules to a mouse brain.^152^ Overall, phase-change agents represent an intriguing vehicle for UTDD applications.

## Other stimulus-responsive delivery methods

Outside of microbubbles, there are also some interesting, emerging devices and systems using cavitation to achieve localized UTDD. One area that has recently gained attention is ultrasound-activated drug delivery devices, whether implantable or transdermal. Delaney *et al.* developed an implantable polymeric reservoir that can be incorporated into existing spinal fusion hardware to provide additional antibiotic prophylaxis following spinal fusion surgery.^51,153^ Briefly, the reservoir is 3D-printed out of polyether ether ketone (PEEK), the hollow inside of the device is filled with a vancomycin solution, and the reservoir is sealed with a thin layer of PLA film. Later, exposure to ultrasound induces cavitation with rupture of the film, which allows for localized release of the encapsulated prophylactic antibiotic at the surgical site.^153^ We have shown that insonation in Doppler mode (1.7 MHz frequency, 6.4 kHz pulse repetition frequency, 100% output power) achieved effective rupture of the PLA film and subsequent release of the vancomycin payload in a cadaveric rabbit model of spinal infection.^153^ Such a device has broad applicability, as the reservoir could be loaded with a variety of drugs or factors. In a slightly different approach, Myers *et al.* designed ultrasound-sensitive polymeric cups for the targeted delivery of oncolytic viruses to cancers.^154^ These cups, comprised of methyl methacrylate, hydroxyethyl methacrylate, and divinyl benzene, delivered the vaccinia virus to HepG2 and SKOV-3 mouse xenografts. Insonation (0.5 MHz, 1.5 MPa peak negative pressure, 5% duty cycle, 10 minutes) induced cavitation of the cups, causing 10 000-fold and 1000-fold enhancement of delivery, respectively.^154^

Thermo-responsive materials have also garnered attention in the field of UTDD. While this review has already discussed the mechanical effects on liposomes, microbubbles, and other agents, this section will focus on the thermal effects of insonation on drug delivery. One of the most commonly studied thermo-responsive vehicles for UTDD is a temperature-sensitive liposome, which locally release their payloads under mild hyperthermic conditions produced by insonation with focused ultrasound.^155^ Aside from liposomes, other thermo-responsive polymers have also been used to create microbubbles and micelles for thermo-responsive UTDD applications.

**Fig. 8:**
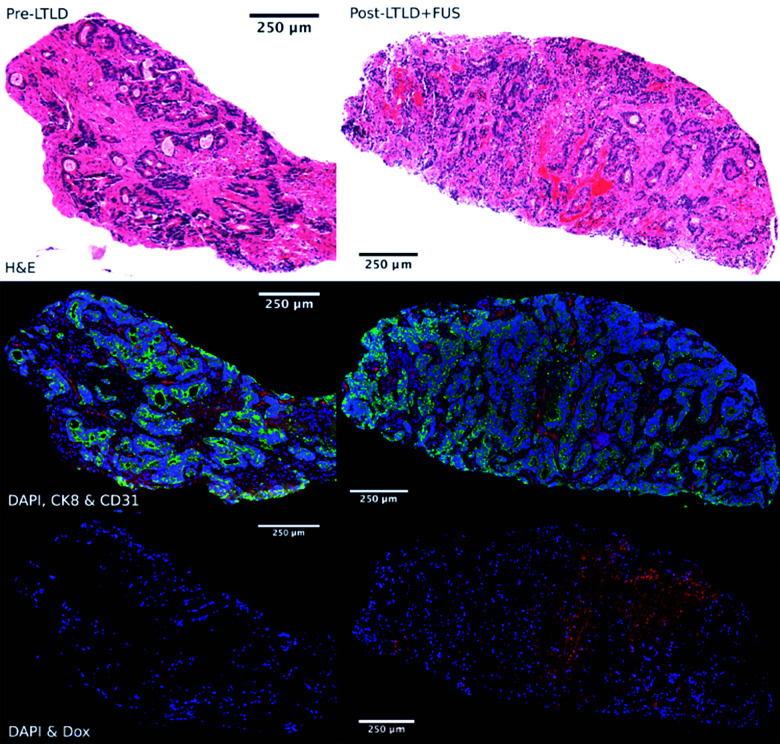
Fluorescence microscopy images of target tumor biopsy samples showing enhanced drug delivery to liver tumors before (left) and after (right) UTDD. Top row: H&E stain demonstrating areas of necrosis within the viable tumor tissue following UTDD. Middle row: Cell viability fluorescence imaging with DAPI (blue), cytokeratin-8 (green), and CD31 (red), showing poor staining in the necrotic areas corresponding with the H&E images. Bottom row: DOX distribution imaging with DAPI (blue) and DOX (red), showing the absence of DOX prior to treatment (left) and nuclear uptake of DOX following insonation (right). Reprinted with permission from ref. 159.

Recently, Huang *et al.* developed microbubbles with a thermo-responsive shell made from poly(*N*-isopropylacrylamide) (PNIPAM).^156^ These microbubbles release gemcitabine under the mild hyperthermia induced by insonation (3 MHz, 2 W cm^−2^, duty cycle of 50%, 1 minute) and have demonstrated therapeutic efficacy (10% apoptosis) in orthotopic pancreatic tumor models in mice, suggesting that this platform could be useful for UTDD in conjunction with thermal ablation of cancerous tumors.^156^ Additionally, Liang *et al.* designed HIFU thermosensitive cerasomes by combining cerasome-forming lipid with conventional lipids (DPPC and DSPE-PEG) to create a drug-carrying vehicle that is highly stable at 37 °C with an 8-times increase in blood circulation time, but releases its payload at 42 °C under HIFU exposure (0.5 MHz, duty cycle 30%, 190 mV, 5 minutes) and provides significant inhibition of MDA-MB-231 breast cancer tumors in mice, with ∼3-fold reduction in tumor volume compared to controls.^157^ However, these mild-hyperthemia-triggered liposomes are highly susceptible to instability during circulation, which can lead to significant off-target release of the drug payload.^155^ Therefore, researchers have begun exploring alternatives, such as liposomes that react in the presence of higher local temperature changes, such as those generated by HIFU. Using commercially available thermosensitive liposomal DOX (ThermoDox), Dromi *et al.* found that 50% of the loaded DOX was released *in vitro* in response to local hyperthermia (42 °C) produced by pulsed HIFU exposure (1300 W cm^−2^, duty cycle 10%, 15–20 minutes), as well as up to 4-fold greater DOX release in an *in vivo* mouse model of breast cancer compared to non-themosensitive liposomal DOX (Doxil).^158^ Excitingly, Lyon *et al.* have published results from a Phase 1 human clinical trial (TARDOX) evaluating the safety and feasibility of UTDD of ThermoDOX for treatment of liver tumors.^159^ Results suggest that their methods were safe, clinically feasible, and importantly, as shown in Fig. 8, effective in enhancing intratumoral drug delivery by almost 4 times following insonation with focused ultrasound (0.96 MHz with duty cycle and power tailored to each patient using a predictive model to achieve 39.5–43 °C at the target site).^159^

Several recent studies have harnessed the various mechanistic advantages of ultrasound to induce or create favorable conditions for drug delivery. For example, Sciurti *et al.* describe the design of PLGA-microPlates for ultrasound-augmented transdermal drug delivery, where the plates are composed of square PLGA microparticles loaded with curcumin.^160^ This group found that application of 1 MHz ultrasound to the microplate resulted in a 200% increase in curcumin release after 30 minutes of insonation, compared to uninsonated controls, suggesting a broad platform for an ultrasound-sensitive matrix filled with drugs for augmented transdermal delivery.^160^ Another system recently designed by Zykova *et al.* used an ultrasound-activated array of polymeric drug-loaded microchambers as a depot system for implant coatings, specifically as an endovascular stent cover.^161^ Using a 1 wt% PLA solution, this group constructed microchamber arrays housing Rhodamine B as a model drug. Exposure of these arrays to low-frequency ultrasound (20 kHz) triggered approximately 55% drug release within 25 seconds.^161^

Ultrasound-mediated drug delivery to the eye has been gaining attention in recent years, with several studies evaluating the safety and efficacy of this technique.^162^ For example, Allison *et al.* demonstrated quadrupled transcorneal delivery of natamycin in an *ex vivo* rabbit cornea model of fungal keratitis using ultrasound (0.5 W cm^−2^, 400 kHz, 5 minutes) compared to controls.^163^ In one of the most recent *in vivo* studies, Nabili *et al.* exhibited almost triple the penetration and delivery of dexamethasone to the aqueous humor of a rabbit using ultrasound (400 kHz, 0.8 W cm^−2^, 5 minutes) compared to passive controls.^164^

While hydrogels were previously discussed as scaffolds for cavitation nuclei, they can also be designed as an acoustically-responsive material.^16^ Kubota *et al.* used solid alginate microbeads (2 wt%), which, when compared to the gaseous microbubbles, have the advantage of superior stability *in vivo*.^16^ To enhance release, these microbeads were also loaded with tungsten particles (0.5–3 μm diameter; 0, 1, and 3 wt%), that, due to their high acoustic impedance, induce vibration within the microbeads. Using this system, release of fluorescent silica nanoparticles increased by up to 30% following insonation (20 kHz, up to 3 minutes). This alternative use of hydrogels for UTDD may be particularly valuable for long term drug release that may go beyond the stability period of microbubbles.^16^

## Conclusions

UTDD impacts the treatment of diseases ranging from Alzheimer's disease to cancer. The power of this approach has become apparent as nano- and microparticle drug delivery has been integrated with the ability to rupture these particles in a focused manner at the site of interest. This external triggering of drug delivery has also been exploited in various materials that respond to diagnostic or therapeutic ultrasound. With the recent advances in use of ultrasound to open up areas previously inaccessible to drug delivery, *e.g.*, delivery through the BBB, the potential of UTDD is offering new methods to release drugs, permeabilize the site of drug delivery, and even allow the drug to reach previously privileged sites.

In this review, we have provided a brief overview of these methods and their dependence on ultrasound-responsiveness of the material properties. Importantly, human trials using a variety of these approaches have recently begun, demonstrating the ability to translate these techniques clinically. As increasingly sophisticated materials are created, their coupling with ultrasound to allow physician-controlled site- and time-dependent triggering offers new methods for non-invasive control of drug delivery.

## List of abbreviations

ACT: Acoustic cluster therapy
ADV: Acoustic droplet vaporization
ARS: Acoustically-responsive scaffold
BBB: Blood brain barrier
DBPC: 1,2-Dibehenoyl-*sn-glycero*-3-phosphocholine
DOX: Doxorubicin
DPPC: Dipalmitoylphosphatidylcholine
DPPE-PEG2000: 1,2-Dipalmitoyl-*sn-glycero*-2-phosphoethanolamine-*N*-[methoxy (polyethylene glycol)-2000]
DSPC: 1,2-Distearoyl-*sn-glycero*-3-phosphocholine
DSPE: 1,2-Distearoyl-*sn-glycero*-3-phosphoethanolamine
DSPE-PEG2000: *N*-(Carbonyl-methoxypolyethyleneglycol 2000)-1,2-distearoyl-*sn-glycero*-3-phosphoethanolamine
DSPG: 1,2-Distearoyl-*sn-glycero*-3-phosphoglycerol
HIFU: High-intensity focused ultrasound
MI: Mechanical index
PDAC: Pancreatic ductal adenocarcinoma
PEG: Polyethylene glycol
PFC: Perfluorocarbon
PLA: Poly(lactic acid)
PLGA: Poly(lactic-*co*-glycolic) acid
UCA: Ultrasound contrast agents
UTDD: Ultrasound-targeted drug delivery
UTMD: Ultrasound-triggered microbubble destruction

## Conflicts of interest

Lauren Delaney, Selin Isguven, and Noreen Hickok do not have any conflicts of interest to report. John Eisenbrey reports equipment, contrast agent, and grant support from GE Healthcare, equipment support from Siemens, contrast agent support and speaker fees from Lantheus Medical Imaging, and royalties from Elsevier. Flemming Forsberg reports equipment support from Canon Medical Systems, GE Healthcare, and Siemens Healthineers, as well as contrast agent support from Bracco, GE Healthcare, and Lantheus Medical Imaging. Flemming Forsberg is a speaker for GE Healthcare and a consultant for Exact Therapeutics.

## Supplementary Material
